# Spontaneous breathing, transpulmonary pressure and mathematical trickery

**DOI:** 10.1186/s13613-020-00708-1

**Published:** 2020-07-08

**Authors:** Luciano Gattinoni, John J. Marini, Mattia Busana, Davide Chiumello, Luigi Camporota

**Affiliations:** 1 Department of Anesthesiology, Emergency and Intensive Care Medicine, University of Göttingen, Göttingen, Germany; 2 Pulmonary and Critical Care Medicine, Regions Hospital and University of Minnesota, St. Paul, MN USA; 3 Department of Anesthesia and Intensive Care, ASST Santi Paolo e Carlo, San Paolo University Hospital, Milan, Italy; 4 Department of Adult Critical Care, Guy’s and St Thomas’ NHS Foundation Trust, and Centre of Human Applied Physiological Sciences, King’s College London, London, UK

Dear Editor,

We read with interest the review by Tobin, Laghi and Jubran on mechanical ventilation in COVID-19 [1]. Rather than a balanced review of the literature, the authors have chosen their sources (mostly opinion pieces) selectively to challenge our interpretation of the data and approach to the problem. Their contention is that patient-self-inflicted lung injury (P-SILI) may be inconsequential to the amplification of lung injury and is not a justification for the ‘liberal use of endotracheal intubation …[which leads to] …fatal complications..’

Having spent large portions of our investigative careers in addressing lung injury and respiratory mechanics, imagine our dismay to learn from them that very few persons require intubation, that P-SILI is a figment of our imaginations, that oesophageal balloons have little value, and that we are using the smoke and mirrors of mathematics to mislead our colleagues. A re-reading of our cited papers has caused us to puzzle why such grave contentions were made by our critics. These deserve a detailed response.

For the reader unaware of the controversial debate on this issue, we summarize our view: patients with COVID-19 acute hypoxaemic respiratory failure (AHRF) often present with profound hypoxaemia paired with unusually good compliance, preserved lung gas volume on CT chest imaging, and substantial increases of respiratory drive and minute ventilation. The excessive drive may amplify the risk of lung damage through P-SILI. If oxygen, HFNC, CPAP, and NIV are unable to subdue vigorous inspiratory efforts even after resolution of hypoxaemia, mechanical ventilation should be applied (i.e., we advocate avoiding delayed intubation—rather than early intubation *per se*). This statement derives from the observation of hundreds of patients in Italy and United Kingdom.

Tobin et al., as a criticism to our approach, maintain that P-SILI is a recent invention, not substantiated by adequate literature [1]. In fact, in 1938 Barach exploited spontaneous breathing to induce experimental lung oedema [2]. Since then, multiple papers in high-tier journals document regional damage from vigorous breathing efforts [3, 4], including a recently published study demonstrating that the median oesophageal pressure swing in patients with moderate or severe AHRF undergoing an NIV trial was 34 cmH_2_O [5]. Reduction in oesophageal pressure swings (DP_es_) was a clear indicator of NIV success and improved chest radiology [5].

In addition, vigorous respiratory efforts increase central blood flow and the likelihood of oedema forming in fluid-permeable lungs. In any case, the argument that the increased tidal volumes seen in heathy pregnant women do not lead to P-SILI cannot be applied to those with injured and diseased lungs. In this context, the study by Mascheroni et al. is cited misleadingly [1, 6]: The primary trigger for VILI is repeated strain associated with excessive transpulmonary pressure, however generated (ventilator or respiratory muscles). Therefore, using the oesophageal pressure swing to quantify the inspiratory effort is not a contributor to “vague and ill-defined concepts, expressed in mathematical terms”. At the pressure we suggested of 15 cmH_2_O, experimental and clinical data indicate that the strain exceeds 1, indicating that tidal volume is, at least, as big as resting lung volume. It is difficult to understand why instituting invasive ventilation when DP_es_ >15 cmH_2_O, admittedly inexact, is equivalent to “playing with fire”. Actually, employing mathematical thresholds to guide treatment is not unknown to the inventor of the rapid shallow breathing index and the advocate of a numerical plateau pressure threshold for VILI. Indeed, the same authors published that DP_es_ is a logical method to monitor weaning, as large DP_es_ are poorly tolerated.

As far as intubation timing, it is far too early to come to a conclusion as to the optimal approach in COVID-19. However, this disease has been characterized by sudden deterioration and lengthy time course [7]. The existing COVID-19 literature reports rates of invasive ventilation ranging from 21 to 90% of all patients with hypoxaemia and ARDS, with mortality rates from 16.7% up to 88–97% of completed episodes [8]. Tobin et al. [9] use this to suggest that invasive ventilation is fatal. However, institutions that adopted an early invasive ventilation strategy have one of the lowest mortality rates reported from the USA. The alternative argument may be that patient selection and a delayed timing of intubation may have played a role. The latter concern has been expressed by Chinese physicians reporting the Wuhan experience [7] and in their expert consensus on COVID-19 [10].

Regarding weaning, we agree with the authors that clinicians often delay extubation. Yet, premature liberation without adequate COVID resolution has led to high reintubation rates (up to 50%). This approach has obvious disadvantages: increased morbidity, mortality and hazard to healthcare staff.

In the end, we thank the authors for their epistemological lesson: finally, we have learned that to prove and disprove something is the basis of scientific progress (Karl Popper would feel gratified). It is possible, then, that future data will disprove the non-existence of spontaneously induced lung injury or prove the tragic consequences of ignoring a growing volume of solid experimental and observational data.
